# A *de novo* deletion mutation in *SOX10* in a Chinese family with Waardenburg syndrome type 4

**DOI:** 10.1038/srep41513

**Published:** 2017-01-27

**Authors:** Xiong Wang, Yaowu Zhu, Na Shen, Jing Peng, Chunyu Wang, Haiyi Liu, Yanjun Lu

**Affiliations:** 1 Department of Laboratory Medicine, Tongji Hospital, Tongji Medical College, Huazhong University of Science and Technology, Wuhan 430030, China; 2 Department of Obstetrics and Gynecology, Tongji Hospital, Tongji Medical College, Huazhong University of Science and Technology, Wuhan 430030, China

## Abstract

Waardenburg syndrome type 4 (WS4) or Waardenburg-Shah syndrome is a rare genetic disorder with a prevalence of <1/1,000,000 and characterized by the association of congenital sensorineural hearing loss, pigmentary abnormalities, and intestinal aganglionosis. There are three types of WS4 (WS4A–C) caused by mutations in endothelin receptor type B, endothelin 3, and SRY-box 10 (*SOX10*), respectively. This study investigated a genetic mutation in a Chinese family with one WS4 patient in order to improve genetic counselling. Genomic DNA was extracted, and mutation analysis of the three WS4 related genes was performed using Sanger sequencing. We detected a *de novo* heterozygous deletion mutation [c.1333delT (p.Ser445Glnfs*57)] in *SOX10* in the patient; however, this mutation was absent in the unaffected parents and 40 ethnicity matched healthy controls. Subsequent phylogenetic analysis and three-dimensional modelling of the *SOX10* protein confirmed that the c.1333delT heterozygous mutation was pathogenic, indicating that this mutation might constitute a candidate disease-causing mutation.

Waardenburg syndrome (WS), also known as auditory pigmentary syndrome, is characterized by congenital sensorineural deafness, dystopia canthorum, and pigmentary abnormalities affecting the hair, skin, and eyes and occurring with a frequency of 1/40,000^1^,^2^. WS is clinically and genetically heterogeneous and is classified into four types (WS1–4) caused by mutations of paired box 3 (*PAX3*), melanogenesis-associated transcription factor (*MITF*), endothelin 3 (*EDN3*), endothelin receptor type B (*EDNRB*), snail-family transcriptional repressor 2 (*SNAI2*), and SRY-box 10 (*SOX10*)^3^,^4^. WS1 and WS2 are the most frequent types, whereas WS4 constitutes a rare disorder^5^,^6^.

WS4 is also known as Waardenburg-Shah syndrome (OMIM 277580) and is characterized by hearing loss, depigmentation, and aganglionic megacolon (Hirschsprung disease). WS4 includes three subtypes [WS4A–C (OMIM 277580, 613265, and 613266)] caused by mutations in *EDNRB, EDN3*, and *SOX10*, respectively^7^,^8^,^9^. Mutations in *EDNRB* and *EDN3* are inherited in the autosomal recessive (AR) or autosomal dominant (AD) form, whereas the *SOX10* mutation is inherited as AD^10^,^11^,^12^,^13^ and found in ~50% of WS4 patients^1^,^6^, with >30 WS4-related mutations reported in the Human Gene Mutation Database. *SOX10* is a critical transcription factor, targeting *MITF*, tyrosinase, myelin protein zero, gap junction protein beta 1, ret proto-oncogene, and *EDNRB* during neural-crest-derived cell migration and differentiation. Additionally, *SOX10* modulates the expression of its target genes and the migration of pluripotent neural crest cells from the neural tube during embryogenesis^14^,^15^.

In this study, we conducted detailed clinical and genetic analysis of a Chinese family with a WS4-afflicted child. A *de novo* heterozygous deletion mutation [c.1333delT (p.Ser445Glnfs*57)] in *SOX10* was detected in the patient, although this mutation was absent in the unaffected parents and 40 ethnicity matched healthy controls. Our findings indicated that this mutation might be a candidate disease-causing mutation.

## Methods

### Subjects and clinical evaluation

The patient, his unaffected parents, and 40 unrelated healthy controls were included in this study, and ophthalmic and audiologic examinations were performed. Written informed consent was obtained from all participants, and this study was formally approved by the Ethics Committee of Tongji Hospital, Tongji Medical College, Huazhong University of Science and Technology. All procedures were performed in accordance with the approved guidelines.

### Mutation screening

Peripheral blood was collected, and genomic DNA was extracted using a DNeasy blood and tissue kit from Qiagen (Hilden, Germany). Polymerase chain reaction (PCR) was performed to amplify all coding exons and intron/exon boundaries of the *EDNRB, EDN3, SOX10, PAX3, MITF*, and *SNAI2* genes. Some of the primers used in the study were referenced from a master’s thesis (title here, Dong Siqi; Chinese PLA General Hospital, Beijing, China), and other primers were designed using Primer 5. Primers are shown in Table 1. PCR of the *SOX10* exons was performed in a total volume of 50 μL containing 60 ng of genomic DNA, 400 nM each of the forward and reverse primers, 40 mM dNTPs, and 2.5 U LA Taq DNA polymerase with GC buffer I from TAKARA (Tokyo, Japan). The amplification consisted of an initial denaturation stage at 94 °C for 3 min, followed by 35 cycles consisting of denaturation at 94 °C for 30 s, annealing for 30 s at 60 °C, and extension at 72 °C for 50 s, with an extension step performed at 72 °C for 3 min. Amplification of exons for the remaining genes was performed using 2× PCR master mix under similar conditions, except for annealing at 57 °C. PCR products were purified and sequenced using an ABI 3500 Dx genetic analyser with a BigDye terminator cycle sequencing ready reaction kit (Applied Biosystems, Foster City, CA, USA), and the sequences were analysed using NCBI BLAST (https://blast.ncbi.nlm.nih.gov/Blast.cgi).

### Paternity testing and haplotype analysis

Five short tandem-repeat markers (STRs; D22S283, D22S1177, D22S1045, D22S272, and D22S423) ranging from chr22:36750705 to chr22:40382524 and five single nucleotide polymorphisms (SNPs; rs139873, rs139885, rs4821733, rs3952, and rs5756908) were selected from the UCSC Genome Browser (http://genome.ucsc.edu/), and linkage-disequilibrium analysis was performed based on LD TAG SNP selection (TagSNP; http://snpinfo.niehs.nih.gov/snpinfo/snptag.php). STR and SNP primers are shown in Table 1.

### Protein structure prediction

Both the wild-type and mutated *SOX10* protein sequences were used to perform protein structure prediction using I-TASSER (http://zhanglab.ccmb.med.umich.edu/I-TASSER/) as previously reported^16^,^17^,^18^,^19^. In I-TASSER, the B-factor, which indicates the extent of the inherent thermal mobility of residues/atoms in proteins, is calculated from threading template proteins from the Protein Data Bank along with sequence profiles derived from sequence databases. The normalized B-factor of the target protein was defined by B = (B′ − u)/s, where B′ represents the raw B-factor value, and u and s represent the mean and standard deviation of the raw B-factors along the sequence, respectively.

## Results

### Clinical findings

**Figure 1:**
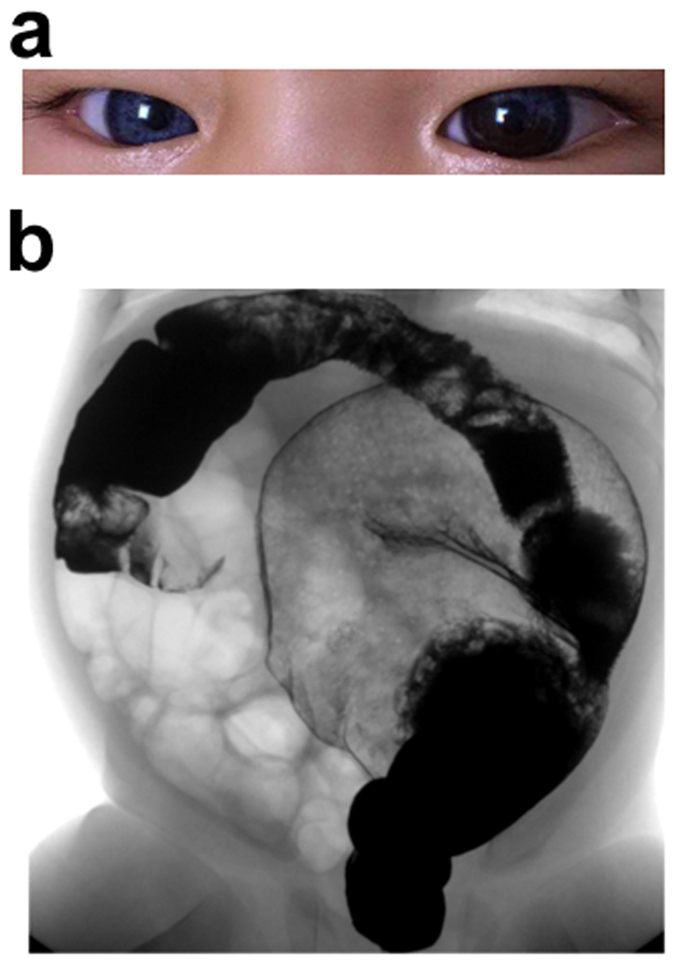
Clinical features of the patient. (**a**) Photograph of the patient presented with blue iride of the right eye and two different colours of the left eye. (**b**) The barium enema examination of the colon of the patient showed megacolon congenitum.

A 1-year-old male patient was referred to our hospital with the chief complaint of Hirschsprung disease accompanied by heterochromia iridis and congenital hearing loss. Based on these clinical features, he was first suspected to be a WS4 patient. Neither parent of the patient exhibited similar symptoms (Fig. 1).

### Identification of a novel *SOX10* heterozygous deletion mutation

**Figure 2:**
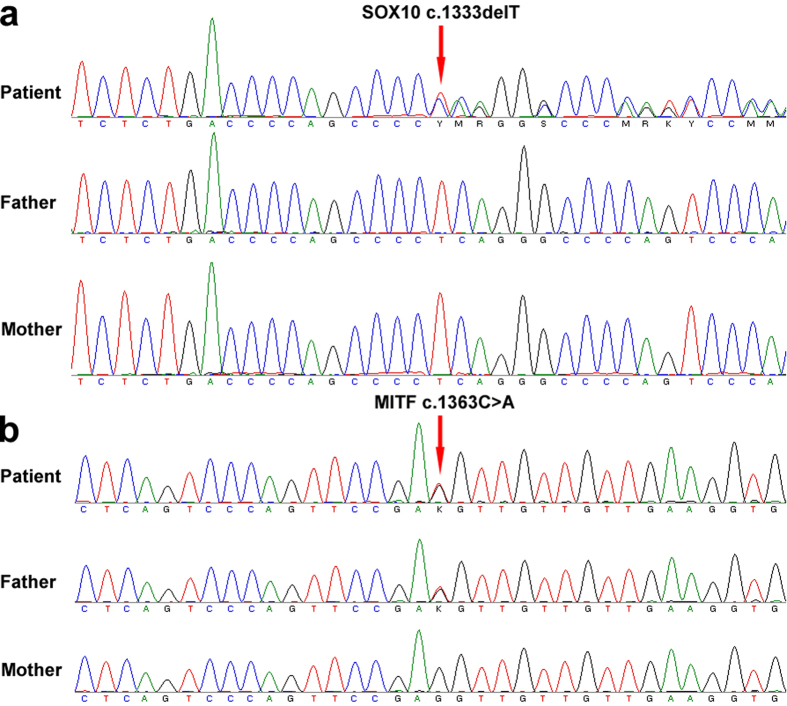
Identification of a novel *SOX10* heterozygous deletion mutation. Sequence chromatographs of the *SOX10* and *MITF* genes of the Chinese family. (**a**) The heterozygous mutation in *SOX10* [c.1333delT (p.Ser445Glnfs*57)] was only found in the patient, but not his father or mother. (**b**) The heterozygosis mutation in *MITF* [c.1363C > A (p.Leu455Ile)] found in the patient and his father, but not his mother.

A heterozygous deletion mutation (c.1333delT) in *SOX10* was identified in the patient, resulting in replacement of the 445^th^ Ser with Gln and a shift in the reading frame to produce a longer protein consisting of 501 amino acids (p.Ser445Glnfs*57) as compared with the wild-type *SOX10* protein (467 amino acids; Fig. 2, Table 2). We subsequently verified that this mutation did not exist in any of the widely used genomic databases, confirming that c.1333delT constitutes a novel deletion mutation. Moreover, this mutation was not found in the unaffected parents or in 40 unrelated healthy control subjects. However, a heterozygous missense mutation (c.1363C > A) in *MITF* was found in both the patient and his father, but not in his mother (Fig. 2). This mutation was found in the dbSNP (https://www.ncbi.nlm.nih.gov/projects/SNP/) and ClinVar (https://www.ncbi.nlm.nih.gov/clinvar/) databases (rs78962087) and is reportedly benign. Furthermore, no mutation was found in the *EDN3, EDNRB, PAX3*, or *SANI2* genes. These results suggested that the heterozygous deletion mutation (c.1333delT) in *SOX10* might be associated with the WS4 phenotype of the patient.

### Paternity testing and haplotype analysis

**Figure 3:**
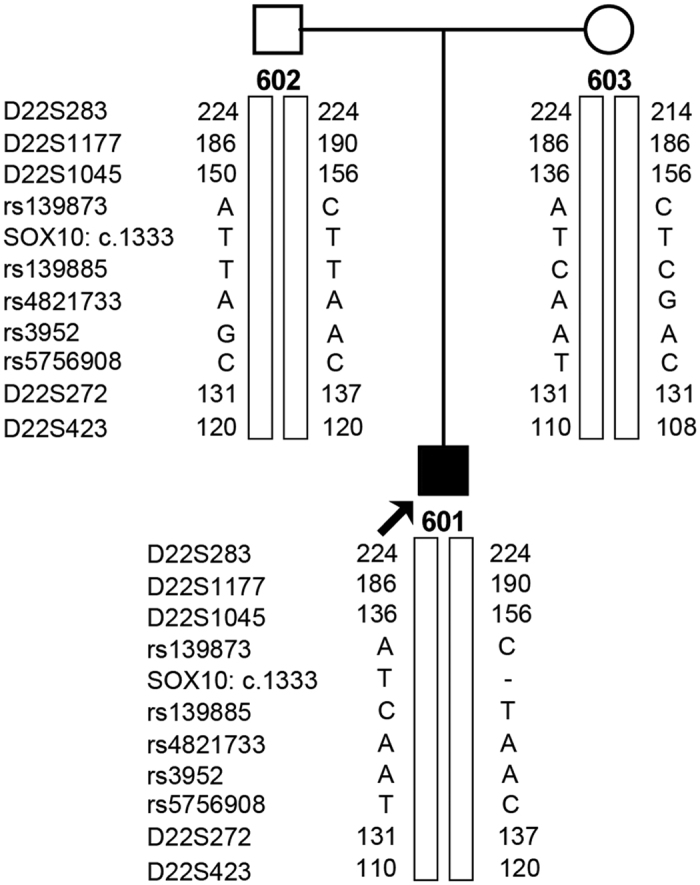
Paternity testing and haplotype analysis. 601, patient; 602, father; 603, mother.

**Figure 4:**
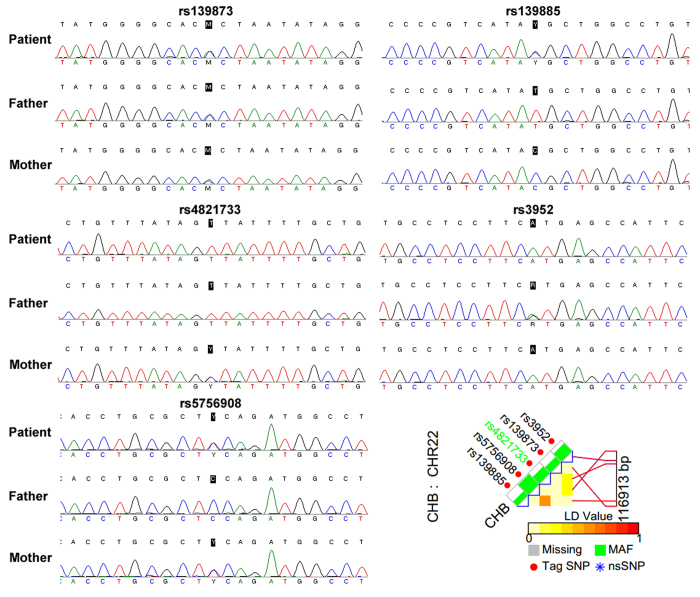
SNP analysis of the Chinese family with WS4. Five SNPs (rs139873, rs139885, rs4821733, rs3952, and rs5756908) were selected.

*SOX10*c.1333delT is located in chr22:38369570. To confirm the paternity of the father, five STRs (D22S283, D22S1177, D22S1045, D22S272, and D22S423) ranging from chr22:36750705 to chr:40382524 and five SNPs (rs139873, rs139885, rs4821733, rs3952, and rs5756908) ranging from chr22:38359666 to chr:38476579 were selected from the UCSC Genome Browser (http://genome.ucsc.edu/) based on their proximity to the mutation site. Paternity testing by haplotype analysis confirmed that these were the biological parents of the patient with WS4 (Figs 3 and 4).

### Protein structure prediction

**Figure 5:**
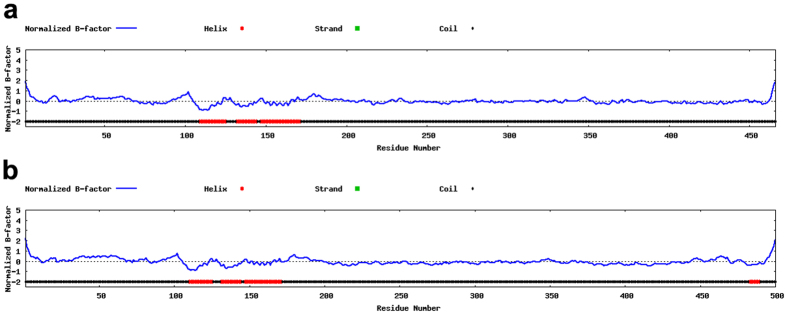
Protein structure prediction. (**a**) Wild-type *SOX10* protein structure. (**b**) The mutated *SOX10* protein structure.

The wild-type *SOX10* protein consists of 467 amino acids and contains three helices, whereas the *SOX10* deletion mutation (c.1333delT) results in a protein consisting of 501 amino acids with four helices (Fig. 5). The wild-type and mutant variants shared identical sequences in the first 444 amino acids, with differences occurring after this point.

## Discussion

WS is classified into four primary phenotypes. WS1 is caused by mutations in *PAX3* and distinguished by the presence of dystopia canthorum (lateral displacement of the inner canthi). WS2 is caused by mutations in *MITF, SOX10*, or *SNAI2* and distinguished from type 1 by the absence of dystopia canthorum. WS3 is caused by mutations in *PAX3,* with patients presenting both dystopia canthorum and upper limb abnormalities. WS4 is caused by mutations in *EDNRB, EDN3*, or *SOX10,* with patients presenting with phenotypes associated with Hirschsprung disease^1^,^20^,^21^,^22^,^23^. Here, we described a Chinese patient with clinical features of WS4 and identified a novel heterozygous deletion mutation [c.1333delT (p.Ser445Glnfs*57)] in *SOX10* that was absent in his unaffected parents and 40 ethnicity matched healthy controls. To the best of our knowledge, this constitutes the first report of this mutation, suggesting it as a candidate disease-causing mutation.

*SOX10* is located on chromosome 22 and encodes an essential DNA-binding nuclear transcription factor consisting of 467 amino acids and belonging to the SOX family involved in modulating embryonic development and determining cell fate. *SOX10* may act as a transcriptional activator upon forming a complex with other proteins and/or as a nucleocytoplasmic shuttle protein critical for neural crest and peripheral nervous system development^24^,^25^,^26^,^27^. Mutations in this gene are associated with WS4 and are present in ~50% of WS4 patients^6^,^28^.

*SOX10* contains a highly conserved high mobility group (HMG) DNA-binding domain and a C-terminal transactivation (TA) domain that is enriched in serine, proline, and acidic residues^29^,^30^. Additionally, *SOX10* contains two separate TA domains, with one localized in the C-terminal region and the other in the central region of the structure. The C-terminal TA domain is frequently involved in various interactions, whereas the TA domain located in the centre of the structure is only involved in TA-related activity in certain cell types and under certain developmental conditions^31^. *SOX10* binds to the promoters of its target genes via the HMG domain, with several studies reporting the importance of the TA domain for inducing transcriptional activation of its target genes^32^. Wang *et al*.^32^ identified a c.1063C > T (p.Q355*) mutation in *SOX10* in a family with WS4 and reported that the mutated *SOX10* variant retained nuclear localization and DNA-binding capabilities comparable to those observed in wild-type *SOX10*; however, the mutated *SOX10* variant was unable to activate transcription of *MITF* via its promoter and acted as a dominant-negative repressor as compared with activity associated with wild-type *SOX10*^7^,^33^. In this study, we detected a c.1333delT (p.Ser445Glnfs*57) mutation in *SOX10* in a family with WS4, with the mutated *SOX10* variant sharing sequence homology with only the N-terminal 444 amino acids of the wild-type protein. Furthermore, we identified an additional helix in the C-terminal region of the mutated *SOX10* variant (Fig. 4), which may affect its normal biological function.

In conclusion, here, we described a *de novo* heterozygous deletion mutation [c.1333delT (p.Ser445Glnfs*57)] in *SOX10* identified in a Chinese family with WS4. Our analyses indicated that this mutation might constitute a candidate disease-causing mutation associated with WS4.

## Additional Information

**How to cite this article**: Wang, X. *et al*. A *de novo* deletion mutation in *SOX10* in a Chinese family with Waardenburg syndrome type 4. *Sci. Rep.* **7**, 41513; doi: 10.1038/srep41513 (2017).

**Publisher's note:** Springer Nature remains neutral with regard to jurisdictional claims in published maps and institutional affiliations.

## Figures and Tables

**Table 1:** Primers used in this study.

Primer name	Sequence
*SOX10* E1F (765 bp)	AGATGGGTTTAGCTGGAGCA
*SOX10* E1R (765 bp)	ACCTGGTCTTCCAGCCCTAT
*SOX10* E2F (686 bp)	GTTATTCCTTGGGCCTCACA
*SOX10* E2R (686 bp)	CTTTGCCCAGTAGGATCAGC
*SOX10* E3A F (686 bp)	GCTGCCAAAATGTGAAACTTA
*SOX10* E3A R (686 bp)	GAGTGGCCATAATAGGGTCC
*SOX10* E3BF (561 bp)	AGCCCAGGTGAAGACAGAGA
*SOX10* E3BR (561 bp)	TCTGTCCAGCCTGTTCTCCT
EDN3 E1 F (407 bp)	CAGAAGCCAGAAAAGCCCGA
EDN3 E1 R (407 bp)	CCAGGCAAGAGTTTGCTCCC
EDN3 E2 F (597 bp)	TTTGCAGACATTTTGCTTGC
EDN3 E2 R (597 bp)	CCTGACCTGCAGAAGAGACC
EDN3 E3 F (480 bp)	GGTGCACAGTTCACTCCAGA
EDN3 E3 R (480 bp)	CCCACAGGACGACAGTAGGT
EDN3 E4 F (607 bp)	CGTCTGTGAAACCCAGTGTG
EDN3 E4 R (607 bp)	CATCACTGCCCAGAGCTACA
EDN3 E5 F (424 bp)	GGCTCGGAAATTGCTGAGAAG
EDN3 E5 R (424 bp)	TCTTTGGGTGGGTGTTCTGC
EDNRB E1 F(748 bp)	CTTTTGAGCGTGGATACTGG
EDNRB E1 R(748 bp)	AGGGAGCTAAAGGGAAGCTC
EDNRB E2 F (498 bp)	AACACACTTTCCTGTCCCATAC
EDNRB E2 R (498 bp)	TTCTACTGCTGTCCATTTTGG
EDNRB E3 F (555 bp)	CTGTGGGAATCACTGTGCTG
EDNRB E3 R (555 bp)	AGCTTGAGTCATTGATCACCA
EDNRB E4 F (432 bp)	TGTTCAGTAAGTGTGGCCTGA
EDNRB E4 R (432 bp)	CAAGAAAAAGGAAATATGCTCTGG
EDNRB E5 F (466 bp)	CACTTCGGTTCCACTTCACA
EDNRB E5 R (466 bp)	CTTCCCTGTCCCTCTCAACA
EDNRB E6 F (493 bp)	GAGGGGGACACAGACAGAGA
EDNRB E6 R (493 bp)	GCAGTAGGGAGTGGCTGACT
EDNRB E7 F (466 bp)	AAGAGGGAAAATAAAAGAGCACTG
EDNRB E7 R (466 bp)	TTCTTTCCATGCCGTAAACA
PAX3 E1F (620 bp)	GAACATTTGCCCAGACTCGT
PAX3 E1R (620 bp)	TCCAAAACAACAGGGACAAGT
PAX3-2F (503 bp)	CCGATGTCGAGCAGTTTCAG
PAX3-2R (503 bp)	CGCACCTTCACAAACCTCAG
PAX3-3F (420 bp)	TGGGATGTGTTCTGGTCTG
PAX3-3R (420 bp)	TCCCAATAGCTGAGATCGA
PAX3-4F (383 bp)	CTGGAGAAGGATGAGGATGT
PAX3-4R (383 bp)	CGTCAGATCACCAATGTCAG
PAX3-5F (508 bp)	TACGGATTGGTTAGACTTGT
PAX3-5R (508 bp)	AACAATATGCATCCCTAGTAA
PAX3-6F (445 bp)	CAACACAGAAGGCAGAGA
PAX3-6R (445 bp)	ATAGGTACGTTCAGGACAA
PAX3-7F (586 bp)	TGTGCAGAGATAGGTGTGAC
PAX3-7R (586 bp)	TTTGATGAAGCCAGTAGGA
PAX3 E8F (543 bp)	GTTATTCTTTCAGCTGTAGGC
PAX3 E8R (543 bp)	GTCTCAACAATTAATAACCGC
MITF E1F (630 bp)	GGAGTTGCACTAGCGGTGTC
MITF E1R (630 bp)	GCTCCATCCGAGCTTCCTA
MITF E2F (628 bp)	GCCTGATAAAAATGCCTTGA
MITF E2R (628 bp)	AGCCACGTAAGAATTAAGGGA
MITF E3F (564 bp)	GCACAGTGCCTGGTACATAAC
MITF E3R (564 bp)	TGCTCTACACCCAATAACCC
MITF E4 F (310 bp)	TCATCTTTTGGTCAGATTCCAC
MITF E4 R (310 bp)	TGCTTAAGTTTTCAGGAAGGTG
MITF-5F (343 bp)	GACCATTATTGCTTTGGGTAAAA
MITF-5R (343 bp)	TGTGATCCTGAGATAATTCTCCATT
MITF-6F (425 bp)	TGAGGAGATCCTGTACCTCTCTT
MITF-6R (425 bp)	AAAAGTTACGTCCATGAGTTGG
MITF-7F (350 bp)	GCTTTTGAAAACATGCAAGC
MITF-7R (350 bp)	GCTGTAGGAATCAACTCTCCTCT
MITF E8 F (527 bp)	AAGGGCTTTGGAAATGGTAA
MITF E8 R (527 bp)	AGAAAGCCACCTCCTCACAA
MITF-9F (425 bp)	CTTATCCATGTAACCAAGCA
MITF-9R (425 bp)	CACACACACAGAATCCACAAA
MITF-10F (646 bp)	CTAATGACGCGCATCTACCA
MITF-10R (646 bp)	TCCTGGGCTATTGATAAAGCA
SNAI2 E1 F (388 bp)	CGGGCTCAGTTCGTAAAGGA
SNAI2 E1 R (388 bp)	GCTCCCTTTCAGGACACTGTTA
SNAI2 E2 AF (534 bp)	GCCCTCCTAAATGGGTCTATC
SNAI2 E2 AR (534 bp)	TTTTCTAGACTGGGCATCGC
SNAI2 E2 BF (565 bp)	GCCCCATTAGTGATGAAGAG
SNAI2 E2 BR (565 bp)	GATCTTTGAGACCAAACCTTC
SNAI2 E3 F (556 bp)	GGTTTTGCTGCTTCTCATTAT
SNAI2 E3 R (556 bp)	TCTCTCAATCTAGCCATCAGC
D22S283 F (217 bp)	FAM-ACAAACACTTCTACAGTCCTGG
D22S283 R (217 bp)	TGAGCCACGGAGATCTTTC
D22S1177 F (186 bp)	FAM-GCCACTCTGGCACCAT
D22S1177 R (186 bp)	AGCTGTNAGCAAGCAGG
D22S1045 F (153 bp)	FAM-GCTAGATTTTCCCCGATGAT
D22S1045 R (153 bp)	ATGTAAAGTGCTCTCAAGAGTGC
D22S272 F (132 bp)	FAM-GAGTTTTGTTTGCCTGGCAC
D22S272 R (132 bp)	AATGCACGACCCACCTAAAG
D22S423 F (123 bp)	FAM-CACACTGGTACACACATACACA
D22S423 R (123 bp)	AAACCAACTGACTCGTTTAGG
rs139885 F (625 bp)	CACCCATGCCTACTGTCTTC
rs139885 R (625 bp)	GAGACCCTGGACCACATACA
rs3952 F (263 bp)	CTTGCTGTAGCCTTGGGAATA
rs3952 R (263 bp)	GTAGAGGGAGGTGGCGAGA
rs5756908 F (306 bp)	AGTTTCCCAAAGATACTGTCCC
rs5756908 R (306 bp)	CCAGTTAGTCCCTCCTCCAA
rs4821733 F (434 bp)	GCAGGCATTGGCATCACC
rs4821733 R (434 bp)	AAATTGCTTGAATGCGGGAG
rs139873 F (374 bp)	AAAAAGACTCCTGGCTTCCA
rs139873 R (374 bp)	CCCACAGTGCTCGGATTC

**Table 2:** Genetic variants found in this family with WS4.

Gene	Variant	Protein level	Type	Father	Mother	Report
*Sox10*	c.1333delT	p.Ser445Glnfs*57	heterozygous	Normal	Normal	No
*MITF*	c.1363C > A	p.Leu455Ile	heterozygous	heterozygous	Normal	Yes
